# Virulence and Replicative Fitness of HIV-1 Transmitted/Founder (T/F) Viruses Harbouring Drug Resistance-Associated Mutation

**DOI:** 10.3390/v16121854

**Published:** 2024-11-29

**Authors:** Aanand Sonawane, Deepak Selvam, Ling Yue, Manohar Nesakumar, Sandhya Vivekanandan, Manickam Ashokkumar, Eric Hunter, Luke Elizabeth Hanna

**Affiliations:** 1 Department of Virology & Biotechnology, ICMR-National Institute for Research in Tuberculosis, Chennai 600031, India; aanandsonawane99@gmail.com (A.S.); smdeepak.s@gmail.com (D.S.); manoicmr@yahoo.co.in (M.N.); sandhya1997sep@gmail.com (S.V.); 2 Department of Immunology, University of Madras, Chennai 600005, India; 3 Department of Pathology and Laboratory Medicine, Emory University, Atlanta, GA 30322, USA; lyue2@emory.edu (L.Y.); ehunte4@emory.edu (E.H.); 4 Department of Medicine, University of North Carolina at Chapel Hill, Chapel Hil, NC 27599, USA; ashokms_86@yahoo.com; 5 Emory Vaccine Center, Emory National Primate Research Center, Atlanta, GA 30329, USA

**Keywords:** HIV, transmitted/founder virus, infectious molecular clones, NNRTI resistance

## Abstract

The biological characteristics of early transmitted/founder (T/F) variants are crucial factors for viral transmission and constitute key determinants for the development of better therapeutics and vaccine strategies. The present study aimed to generate T/F viruses and to characterize their biological properties. For this purpose, we constructed 18 full-length infectious molecular clones (IMCs) of HIV from recently infected infants. All the clones were characterized genotypically through whole genome sequencing and phenotypically for infectivity, replication kinetics, co-receptor usage, as well as their susceptibility to neutralizing antibodies and entry inhibitors using standard virological assays. Genotypic analysis revealed that all the T/F clones were of non-recombinant subtype C, but some of them harboured the Y181C drug resistance mutation associated with resistance to the non-nucleoside reverse transcriptase inhibitor (NNRTI) class of antiretroviral drugs. In vitro studies showed that while all the IMCs were capable of replicating in PBMCs and utilized the CCR5 co-receptor for cellular entry, the drug-resistant variants had significantly lower replicative capacity and per particle infectivity than the drug-sensitive viruses. Both exhibited similar sensitivities to a standard panel of broadly neutralizing monoclonal antibodies and viral entry inhibitors. These findings suggest that despite their diminished replicative fitness, the drug-resistant T/F variants retain transmission fitness and remain susceptible to neutralizing antibody-based interventions and viral entry inhibitors.

## 1. Introduction

Globally, approximately 1.3 million women and girls living with HIV become pregnant each year (WHO, 2022) [1]. Without intervention, the risk of HIV transmission from a HIV-positive mother to her child during various stages of pregnancy is estimated to be 15–30% during gestation or labor and 15–20% through breastfeeding [2]. Various interventions are now being employed to prevent mother-to-child transmission (PMTCT) of the virus [3]. However, the effectiveness of PMTCT strategies may be compromised by the emergence of drug-resistant HIV strains that can potentially be transmitted from the mother to the child despite the use of antiretroviral therapy (ART) administered for PMTCT [3,4].

HIV exhibits significant genetic diversity due to the error-prone nature of the viral reverse transcriptase (RT), leading to the emergence of a complex quasispecies in chronically infected individuals. However, it is evident that a genetic bottleneck occurs during HIV transmission, allowing only a select few genetic variants to establish infection in the newly infected host. Understanding the unique characteristics of the transmitted/founder (T/F) virus, i.e. the viral strain that initiates HIV-1 infection, is crucial for the design of effective immune-based or other prophylactic interventions against HIV. Detailed biological characterization of T/F viruses has been challenging due to the limited availability of full-length infectious molecular clones (IMCs) of these variants. In this study, we aimed to develop IMCs of T/F viruses from infants recently infected with HIV through vertical transmission from their mothers. Incidentally, we found that some of the T/F clones that we generated carried a mutation in the reverse transcriptase (RT) gene that conferred resistance to the non-nucleoside reverse transcriptase inhibitor (NNRTI) class of antiretroviral drugs used for the treatment of HIV as well as for pre-exposure prophylaxis (PrEP) in pregnant women and their children [5].

NNRTI drugs bind to a hydrophobic pocket located near the polymerase active site and effectively prevent the synthesis of viral DNA [6,7]. The administration of a single dose of nevirapine (NVP) to both the delivering woman and the neonate within 72 h of birth has been shown to be a safe and effective measure for preventing mother-to-child transmission of HIV [8,9]. However, it is now well known that nevirapine exposure selects viral mutations associated with resistance to NNRTIs in more than half of exposed infants [10]. K103N and Y181C substitutions are the two most common NNRTI-resistance associated mutations. Several studies have shown that whereas K103N is the predominant NNRTI-resistant mutation in adults, Y181C is the predominant NNRTI-resistant mutation among infants [11]. The Y181C mutation in the reverse transcriptase (RT) enzyme of HIV involves the substitution of a tyrosine residue with a cysteine (Y181C) in the NNRTI binding pocket [12]. This mutation confers high level resistance to drugs like nevirapine (NVP) and delavirdine (DLV), and moderate resistance to etravirine (ETR) and rilpivirine (RPV) [13,14].

Mutations that confer drug resistance are known to reduce the ability of HIV-1 to replicate effectively [15,16]. Yet, the transmission of drug-resistant variants is known to occur as evidenced by the presence of T/F viruses carrying resistance-associated mutations in recently infected infants [17,18]. In addition to generating full-length patient-specific infectious clones of T/F viruses from recently infected infants, the present study also attempted to characterize the impact of a high-level drug resistance-associated mutation in the T/F clones on replicative fitness, viral infectivity, co-receptor usage, and sensitivity to broadly neutralizing antibodies (bNAbs).

## 2. Materials and Methods

### 2.1. Study Subjects

The current study used stored plasma samples of two HIV-1-infected infants who had recently contracted the infection through mother-to-child transmission. The HIV status of the infants was confirmed in the laboratory using HIV-1 DNA PCR. Table 1 presents the comprehensive demographic profile of the two infants. The samples were collected with the approval of the Institutional Ethics Committee of the ICMR-National Institute for Research in Tuberculosis (NIRT-IEC 2021 038).

### 2.2. Viral RNA Extraction and cDNA Synthesis

Viral RNA was extracted from plasma using the QIAamp viral RNA mini kit from Qiagen (Hilden, North Rhine-Westphalia, Germany) following the manufacturer’s instructions. In brief, 140 μL of plasma was used for RNA extraction. The extracted viral RNA was immediately converted to near-full-length cDNA (FLcDNA) using Superscript IV reverse transcriptase enzyme from Life Technologies and in-house primers.

### 2.3. PCR Amplification of near Full-Length Viral Genome

We used the single genome amplification (SGA) approach to amplify near full-length viral (NFL) genomes of approximately 9 kb using the in-house primers listed in Table 2, following the protocol described by Deymier et al. [19]. Briefly, the cDNA synthesized from viral RNA was serially diluted and distributed into ten replicate wells of a PCR plate, so as to reach a dilution point at which 30% of the wells exhibited positive amplification [20,21]. This dilution threshold was determined to ensure that each well contained a single viral template. This was followed by nested PCR using HIV-specific primers. First-round PCR was carried out in a total volume of 50 μL with a reaction mixture comprising of 1X KAPA HiFi premix (Roche, Kapa Biosystems, Cape Town, South Africa) and 0.5 μM each of the primers HCFLC1F and HCFLC1R (Oligo primers synthesize from IDT Technologies, Coralville, IA, USA). The first round of PCR comprised of initial denaturation at 98 °C for 30 s, 35 cycles of denaturation at 98 °C, annealing at 66 °C, and extension at 72 °C for 10 s, 30 s, and 8 min, respectively, and final extension at 72 °C for 10 min. The second round of PCR was performed with the same cycling conditions using the same PCR master mix as round 1, with 2 µL of the first-round PCR product as template and HCFLC2F and HCFLC2R as primers. The amplicons were analysed by gel electrophoresis to confirm the presence of the expected 9 kb product.

### 2.4. LTR Amplification and Construction of Patient-Specific LTR Clone

The virion-packaged RNA genome of retroviruses does not contain the complete long terminal repeat (LTR) region [22]. In order to construct full-length infectious molecular clones from the amplified near full-length HIV-1 genomes, we separately amplified 650 bp of the LTR sequence from the genomic DNA of the corresponding infant using nested PCR, with the LTR CF and LTR CR primers for the first round and LTR overlap vec F and LTR overlap vec R primers for the second round. The PCR reaction was performed in a total reaction volume of 50 μL containing 1X KAPA HiFi (Roche, Cape Town, South Africa) reaction premix and 1 μL of DNA template. The cycling conditions included an initial denaturation step at 98 °C for 2 min, followed by 35 cycles of denaturation at 98 °C for 20 s, annealing at 62 °C for 20 s, and extension at 72 °C for 1 min, with a final extension at 72 °C for 2 min. Two microlitres of the first-round PCR product was used as template for the second-round PCR. The total volume of round II PCR was 50 μL with similar components as the first round, except for the primers, which had an overlap with the vector. The amplicons were visualized on a 1% agarose gel. Positive bands were excised using a gel cutter, DNA was extracted using the Qiagen gel extraction kit (Qiagen, Hilden, North Rhine-Westphalia, Germany)and eluted in 60 μL of nuclease-free water.

To generate patient-specific LTR clones, we generated a linear vector amplicon from pIndie, a HIV-1 subtype C molecular clone which contains a bacterial origin of replication and ampicillin resistance cassette. The primers used for the amplification of the vector were Pvec F and Pvec R. The PCR conditions used were initial denaturation at 98 °C for 30 s, followed by 35 cycles of 98 °C for 20 s and 72 °C for 4 min, with a final extension at 72 °C for 7 min. The 3.8 kb vector piece was gel-extracted and purified. The patient-specific LTR amplicons and vector backbone were mixed in a ratio of 1:1. Two microliters of In-Fusion HD 5X enzyme premix was added, and the reaction volume was made up to 10 μL with nuclease-free water. The reaction mixture was incubated at 50 °C for 45 min and then held at 4 °C until it was ready for transformation.

The LTR-pIndie vector infusion reaction mix was transformed into competent Stellar cells following the manufacturer’s protocol (Clontech, CA, USA). In brief, the competent cells were thawed on ice. Twenty-five microlitres of competent cells was transferred to a 1.5 mL microcentrifuge tube and placed on ice, and 2–3 μL of the In-Fusion HD cloning reaction (Takara, San Jose, CA, USA) mix was added to it. The mixture was incubated on ice for 30 min. The cells were heat shocked for 45–60 s in a water bath and quickly placed on ice for at least for 2 min. Next, 500 μL of warm SOC medium was added and incubated at 37 °C for 30 min with gentle shaking at 180 rpm in a shaker. Then, 100 μL of cells was plated on LB agar plates supplemented with 100 μg/mL ampicillin (Sigma-Aldrich, Saint Louis, MO, USA) and incubated at 37 °C overnight. Multiple colonies were picked and cultured overnight in LB broth. DNA was extracted using the PureYield Plasmid Miniprep System (Promega, Madison, WI, USA). The LTR vector clones were confirmed by double restriction digestion and sequencing of the LTR-pIndie vector junction using Sanger sequencing.

### 2.5. Generation of Patient-Specific Full-Length Infectious Molecular Clones (IMCs)

To assemble full-length HIV clones, the patient-specific LTR vector clone was used to generate two individual PCR products as per an established protocol described previously for the production of full-length molecular clones [19]. This process was meant to fill the absent LTR regions from the NFL amplicons in the construct. Fragment one was amplified in a 50 μL reaction with 1X KAPA premix (Roche, Cape Town, South Africa), 5′ LTR VECF, and 5′ LTR VECR as primers. The PCR reaction included an initial denaturation step at 98 °C for 30 s, 30 cycles of denaturation at 98 °C, annealing at 60 °C, and extension at 72 °C for 10 s, 30 s, and 2 min, respectively, followed by a final extension at 72 °C for 3 min.

Similarly, the second vector fragment was amplified using the same reaction mixture, with primers 5′ LTR VECF and 3′ LTR VECR. The cycling conditions used were initial denaturation at 98 °C for 30 s, followed by 30 cycles of denaturation at 98 °C for 10 s, annealing at 67 °C for 30 s, extension at 72 °C for 2 min, and final extension at 72 °C for 3 min. For infusion cloning of the full-length HIV genome, the 9 kb NFL amplicon was derived by re-amplification of the selected second-round amplicon using the cloning primers NFL C clone F and NFL C clone R. The PCR conditions were the same as described previously for the NFL PCR, except for the cycling conditions. The cycling conditions included an initial denaturation step at 98 °C for 30 s, 30 cycles of denaturation at 98 °C, annealing at 65 °C, and extension at 72 °C for 10 s, 30 s, and 8 min, respectively, followed by a final extension for 10 min at 72 °C.

Each of the three PCR products were individually gel-extracted as described earlier. To generate the full-length clone, a final In-Fusion HD reaction was performed. In a 10 μL reaction volume, the 9 kb NFL amplicon was combined with the two LTR vector pieces that complemented the missing LTR regions. The reaction included 150 ng of each linear gel-purified amplicon, 2 μL of 5X In-Fusion HD enzyme (Takara, CA, USA) mix, and nuclease-free water. The reaction mixture was incubated at 50 °C for 15 min and then kept at 4 °C until transformation.

Stellar competent cells were transformed with the In-Fusion HD reaction mixture. Multiple colonies were picked and cultured for plasmid DNA isolation. Recombinant clones were identified using restriction digestion. The selected clones were grown in 250 mL of LB broth containing ampicillin, and plasmid DNA was extracted using the Pure Yield Plasmid Maxiprep System (Promega, Madison, WI, USA) and used for virus production for molecular and phenotypic characterization.

### 2.6. Generation of Virus Stocks and Determination of Infectivity

Virus stocks were produced by transfecting 293T cells with plasmid DNA containing full-length HIV-1 genomes, following a previously described protocol [19,23]. In brief, around 2 × 10^5^ HEK 293T cells were seeded in each well of a 12-well plate one day prior to transfection. On the subsequent day, 2 μg of individual plasmid DNA was transfected into the cells using the FuGENE HD reagent (Promega, Madison, WI, USA), following the manufacturer’s instructions. After 48 to 72 h of transfection, culture supernatants were collected, clarified through low-speed centrifugation, and stored at −80 °C. The infectivity and 50% infectious dose (TCID_50_) of the virus stocks were determined by infecting TZM-bl cells using a previously described protocol [24,25]. In brief, TZM-bl cells were seeded in a 96-well plate with 100 μL of complete DMEM. The viral stocks were added to quadruplicate wells in the first column of the plate and serially diluted across the wells in the remaining columns. After 48 h, 100 μL of the culture medium was removed from each well and 50 μL of passive lysis buffer was added. The plates were incubated for 2 min at room temperature for cell lysis. The cell lysate was transferred to a flat-bottom, black, solid, 96-well plate for the measurement of luciferase activity using the Bright-Glo luciferase assay reagent (Promega, Madison, WI, USA). The plates were read using an Infinite M200Pro plate reader (Tecan, Männedorf, Switzerland), and the results were expressed as relative light units (RLU). Clones that were infectious were selected for further analysis.

### 2.7. Sequence Analysis and Drug Resistance Genotyping

Full-length infectious clones were sequenced on the Illumina NGS platform(Illumina NextSeq500, San Digeo, CA, USA). Sequences were analysed using the Geneious software, v10.0.9. Sequence contigs were assembled to generate the complete genome sequences using full-length HIV-1 subtype C reference sequences from the Los Alamos Database (https://www.hiv.lanl.gov/cgi-bin/NEWALIGN/align.cgi, accessed on 1 August 2022) as the reference. HIV subtyping analysis was performed using both the Recombination Identification Program (http://www.hiv.lanl.gov/content/sequence/RIP/RIP.html, accessed on 4 July 2022) and the Rega HIV subtyping tool (https://www.genomedetective.com/app/typing_tool/hiv, accessed on 5 July 2022). HIV-1 Pol sequences were submitted to the Stanford University HIV Drug Resistance Database (HIVdb program, version 6.0.11 at http://hivdb.stanford.edu) to screen for the presence of drug resistance-associated mutations.

### 2.8. Identification of Early Transmitted/Founder Viruses

HIV-1 transmission at the mucosal surfaces is characterized by a stringent bottleneck, typically resulting in the successful establishment of infection by just one or few variants out of the quasispecies, referred to as transmitted/founder (T/F) variants, which leads to productive clinical infection in most cases [26]. We analysed the sequences of all the infectious full-length clones obtained from each of the two infants using neighbour-joining (NJ) phylogenetic trees constructed using MEGA7 and highlighter plots generated using the online highlighter tool available at www.hiv.lanl.gov (accessed on 1 August 2022) [27,28,29]. These tools help in tracing common ancestry between sequences by identifying individual nucleotide polymorphisms. T/F variant lineages were identified based on the following three criteria: firstly, branches containing >2 identical sequences; secondly, sequences that clustered closely on the same branch; and thirdly, individual sequences that did not contain any recombination signature.

### 2.9. Analysis of In Vitro Replication in Peripheral Blood Mononuclear Cells

We assessed the replication kinetics of the Y181C mutant and non-mutant T/F viruses using CD8 T cell-depleted peripheral blood mononuclear cells (PBMCs). CD8 depletion was achieved using anti-CD3/8 bi-specific monoclonal antibody (at 50 μg/mL; final concentration 0.5 μg/mL, 1/100 of CD3/8) following a previously described method [30]. The CD8-depleted PBMC were infected with an MOI of 0.05 of virus in triplicate wells. After 3 h of incubation at 37 °C, the plate was centrifuged to remove the unbound virus. The washed cells were resuspended in 1 ml of RPMI-1640 medium containing 10% FBS and 20 U/mL of IL-2. The cell suspension was then transferred to a 48-well plate and incubated at 37 °C in a 5% CO_2_ incubator. Five-hundred-microlitre aliquots of the culture supernatant were harvested on days 0, 2, 4, 6, 8, and 10, and the volume was replenished with fresh medium. Virus production was estimated in terms of reverse transcriptase activity in the harvested culture supernatants following previously established methods [30,31]. The replication score (RC score) for each variant was determined by calculating the normalized area under the curve. The median of the replicates was background-subtracted using the day 2 time point, adjusted for sampling through a measured exponential decay correction. The area under the curve (AUC) was then divided by the AUC for a standard lab-adapted subtype C virus, MJ4. This normalization allowed for a comparison across transmission pairs that were analysed on different days.

### 2.10. Determination of Per Particle Infectivity

The per particle infectivity of the generated clones was assessed using a single-round infection assay as described previously [19,23]. In summary, TZM-bl cells (1 × 10^5^ cells) were individually seeded and cultured in wells of a 96-well plate with 100 μL of DMEM growth medium per well, each in quadruplicate. On the following day, the cells were exposed to approximately 200 TCID_50_ of each viral variant, resulting in a multiplicity of infection (MOI) of 0.02. Following 6 h of incubation at 37 °C, the infected cells were washed twice with culture medium and incubated at 37 °C for an additional 48–72 h. After this, the infected cells were lysed, and viral infectivity was assessed by measuring luciferase activity in the lysate in terms of relative light units (RLU) using the Bright-Glo luciferase assay system and Infinite M200pro plate reader. To ensure the reliability of the results, all experiments were performed in triplicates.

### 2.11. Determination of Co-Receptor Usage

To determine the co-receptor usage and tropism of the viral clones, we cultured TZM-bl cells with and without a CCR5 inhibitor (Maraviroc) and a CXCR4 inhibitor (AMD3100) at a concentration of 2 μM. A total of 200 TCID_50_ of the virus was added to each well, and the cells were cultured for 48 h [32,33]. After 48 h of culture, the cells were stained with a β-gal substrate and the number of blue cells was counted to determine infectivity in the presence and absence of the co-receptor inhibitors.

We also performed co-receptor analysis using GHOST cells that express specific entry receptors for HIV such as CXCR4+ and CCR5+ cells. GHOST cells contain an LTR promoter cassette that expresses green fluorescent protein (GFP) in response to HIV infection. One million GHOST cells expressing each of the above co-receptors were cultured in 12-well plates separately. After 24 h of culture, the cells were infected with the virus at an MOI of 0.05 in the presence of 10 μg/mL DEAE. The cells were analysed for GFP expression after 72 h on a FACS Canto II (Becton Dickinson Biosciences, Eysins, Switzerland) flow cytometer.

### 2.12. Determination of Sensitivity to Maraviroc

The sensitivity of the drug-sensitive and drug-resistant T/F clones to the antiretroviral drug maroviroc (MVC) was assessed as per previously described methods [23,34]. In brief, 1 × 10^5^ TZM-bl cells were infected with viruses exposed to different concentrations of the CCR5 antagonist, starting from 10^1^ µM to 10^−5^ nM, for 2 h prior to infection with 0.05 MOI of the virus. Untreated culture without drug exposure served as the negative control. Forty-eight hours post-infection, the cells were lysed with 1X passive lysis buffer (Bright-Glo luciferase assay system, Promega, Madison, WI, USA), and luciferase activity was measured in terms of relative light units using the Infinite M200pro plate reader(Tecan Trading, Männedorf, Switzerland). The half-maximal effective concentration (EC_50_) of MVC was determined using non-linear regression analysis (GraphPad Prism, version 8.0.1).

### 2.13. Determination of Sensitivity to Broadly Neutralizing Antibodies (bNAbs)

To determine the intactness of the viral envelopes, we tested the neutralization sensitivity of the viral isolates to broadly neutralizing antibodies (bNAbs) using a panel of well-characterized bNAbs like VRC01, PG16, 10E8, PG128, and PG06 that target distinct regions on the HIV-1 envelope. The neutralization assay was performed using different concentrations of bNAbs ranging from 10 µg/mL to 0.001 µg/mL. A total of 200 TCID_50_ of the virus that was pre-incubated with the respective monoclonal antibody (mAb) at 37 °C for 2 h was used to infect 1 × 10^5^ TZM-bl cells seeded in a flat-bottom 96-well plate. After 48 h, luciferase activity was measured in terms of relative light units using a luminometer (Perkin Elmer, MN, USA)and the IC_50_ value was calculated using GraphPad Prism 8.0.1. Concentrations falling outside the range of the tested dilutions were reported as maximum or minimum values accordingly.

### 2.14. Phenotypic NNRTI Susceptibility Testing

NNRTI drug susceptibility testing was performed to assess the sensitivity of the Y181C mutant and non-mutant T/F clones to other NNRTI drugs like efavirenz (EFV), etravirine (ETR), nevirapine (NVP), and rilpivirine (RPV) (NIH-AIDS Reagent Programme, USA). In brief, TZM-bl cells were seeded in a 96-well plate at a concentration of 1 × 10^5^ cells per well [35,36]. Serial dilutions of the drugs were added to the cells. The cells were then infected with the virus at an MOI of 0.05. After 48 h of incubation under controlled conditions at 37 °C in a 5% CO_2_ incubator, the cells were lysed and the relative light units (RLU) per well were quantified using the Wallac 1420 luminometer (Perkin Elmer, Waltham, Massachusetts, USA). The IC_50_ value (concentration that resulted in a 50% reduction in viral activity for each NNRTI and viral strain) was calculated using GraphPad Prism 8.0.1.

## 3. Results

### 3.1. Construction of Full-Length Molecular Clones of Recently Transmitted/Founder Viruses Using the In-Fusion HD Cloning Technique

**Figure 1:**
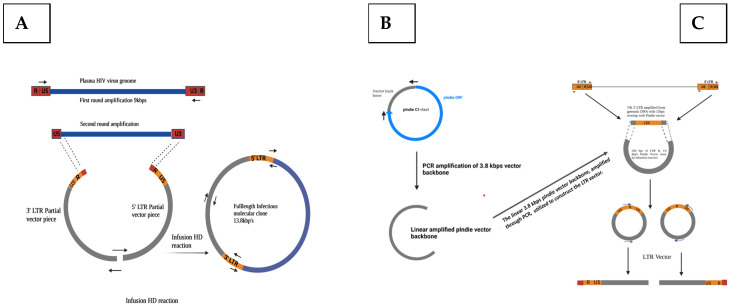
**Full-length genome cloning strategy.** (**A**) The figure illustrates the amplification 3.8 kbps vector backbone from the pIndie HIV-1 subtype C clone. (**B**) LTR was amplified from the PBMC DNA of the infants and cloned using the In-Fusion cloning system. The 4.5 kb LTR vector was employed to generate two individual PCR products for the final cloning reaction, encoding the missing LTR regions from the full-length genome amplicon. (**C**) A previously amplified 9 kb near full-length HIV genome was used as a template for re-amplification with an overlapping primer set which had 15 bp homology with the two LTR_Vec fragments. In the final cloning step, the 9 kb full-length genome amplicon was cloned with the 5′ LTR_vec fragment and 3′ LTR_vec fragment that share the homology sequence for the infusion reaction to generate full-length infectious clones.

Our cloning strategy (Figure 1) aimed to amplify and clone the full-length HIV-1 genome from viral RNA present in the plasma of two acutely infected infants who acquired the disease from their respective mothers (Table 1). We employed single genome amplification (SGA) to amplify the near full-length (NFL) HIV-1 genome from plasma viral RNA using the primers listed in Table 2. Since the plasma virus genome typically lacks a complete LTR, we performed a separate PCR to amplify a 650 bp LTR fragment from genomic DNA isolated from the corresponding infant’s PBMC. This approach ensured accurate representation of the virus sequence by specifically targeting and matching the LTR. Patient-specific LTR primers were used for nested PCR based on the identified sequence at the 5′ end of U3 and 3′ end of U5 in the NFL amplicon. Cloning of the LTR fragments was achieved using the pIndie vector backbone via a two-piece fusion reaction. Subsequently, PCR reactions were performed to generate 5′ and 3′ LTR fragments, followed by re-amplification of the NFL genome with homology to both LTR vector fragments. Finally, the three pieces (5′ LTR fragment, 3′ LTR fragment, and NFL genome amplicons) were combined in an In-Fusion HD cloning reaction to produce full-length molecular clones of the T/F virus. Several full-length clones were analysed for infectivity using the standard TZM-bl assay.

### 3.2. Infectivity of the Constructed Full-Length T/F Clones

Virus stocks were produced from each molecular clone by the transfection of 293T cells. Infectivity of the virus stocks was tested using the standard TZM-bl assay. After screening a large number of clones, we identified 13 infectious clones belonging to sample 822 and 5 infectious clones belonging to sample JUS. All the 18 full-length infectious clones were subjected to whole genome sequencing on the Illumina platform.

### 3.3. Sequence Analysis and Identification of Early Transmitted/Founder Viruses

**Figure 2:**
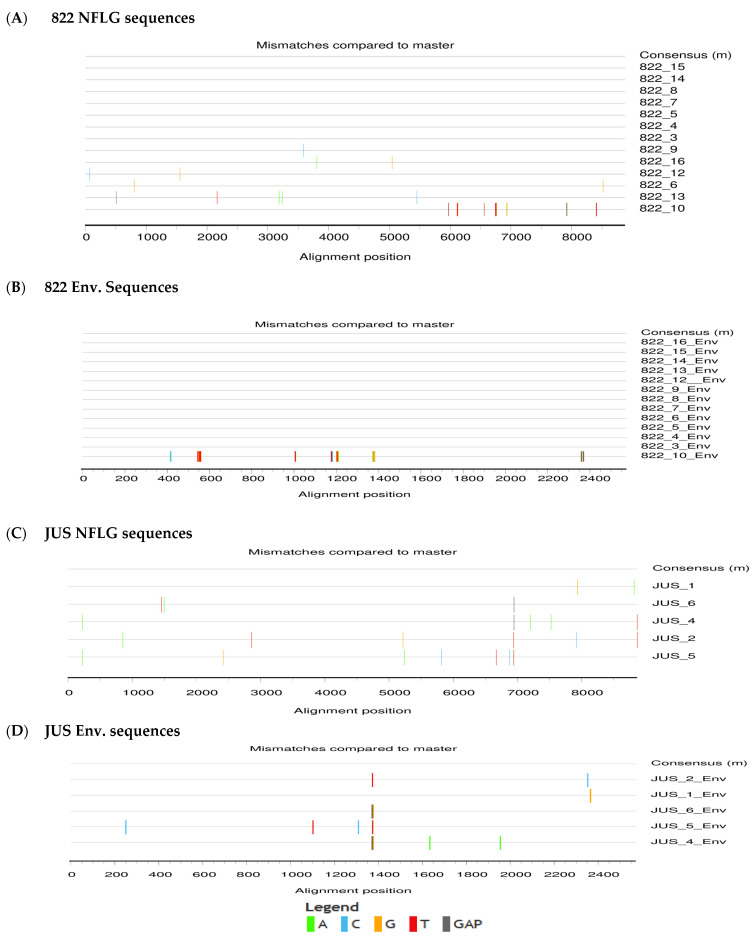
**Highlighter plot of HIV-1 full-length genome and envelope sequences of the molecular clones.** (**A**,**B**) Full-length genome and envelope sequences of sample 822; (**C**,**D**) Full-length genome and envelope sequences of sample JUS.

The sequences of the full-length infectious clones were found to be low-diversity monophyletic sequences, with sequences derived from each infant forming a single lineage in the NJ tree. The highlighter plots also revealed very low level of genetic diversity, indicating minimal viral evolution during the short period post-infection. This is because both the infants were very recently infected with HIV, i.e., 38 days and 22 days after birth for 822 and JUS, respectively. Highlighter plots indicating the positions and identities of nucleotide polymorphisms, insertions, and deletions across the genomes are shown in Figure 2. Among the 13 IMCs obtained from sample 822, 12 (92.3%) had envelope sequences similar to the consensus sequence. Out of the five clones obtained from sample JUS, three sequences aligned well with its consensus sequence.

Interestingly, sequence analysis revealed the presence of an NNRTI drug resistance-associated mutation, Y181C, in the T/F clones obtained from one infant (822), while the clones from the other infant (JUS) did not have any drug-resistance-associated mutation. Upon further investigation, it was identified that the mother of the infant whose virus carried the Y181C mutation also harboured viruses with the same mutation.

### 3.4. In Vitro Replication Capacity of the T/F Viruses

**Figure 3:**
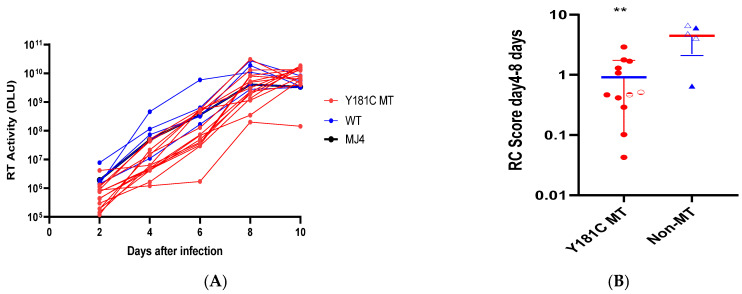
**In vitro replication capacity of Y181C mutant and non-mutant (WT) viruses in PBMC.** (**A**) Virus growth was monitored over 10 days at 2-day intervals in PBMC cultures, by measuring reverse transcriptase (RT) activity in digital light units (DLU) for the Y181C mutant (red) and non-mutant variants (blue). MJ4 virus (black) was used as the control. (**B**) The replicative capacity (RC) scores were determined by calculating the area under the curve relative to MJ4 for all tested viruses. The RC score for the Y181C mutants was notably lower compared to the non-mutants (** *p* = 0.0068). The Y181C mutant and non-mutant variants are represented by the red circle and blue triangle, respectively.

We sought to assess the replication potential of the generated T/F clones on primary cells and, at the same time, determine the impact of the Y181C mutation on the replicative ability of the reverse transcriptase gene of the 822 clones. For this purpose, we performed a replication kinetics assay using CD8 T cell-depleted primary PBMC obtained from a HIV-seronegative donor. Virus growth was monitored by measuring reverse transcriptase activity in cell culture supernatants at 48 h intervals over ten days (Figure 3A). Viral replication capacity (VRC) scores were determined by calculating the area under the curve for each virus, normalized with the MJ4 HIV-1 clone. We observed that the RC score of the mutant T/F viruses was significantly lower than that of the non-mutant viruses (*p* = 0.006, Figure 3B). We also used a TZM-bl assay to confirm that the viruses produced were infectious (Supplementary Figure S1).

### 3.5. Per Particle Infectivity of the T/F Clones

**Figure 4:**
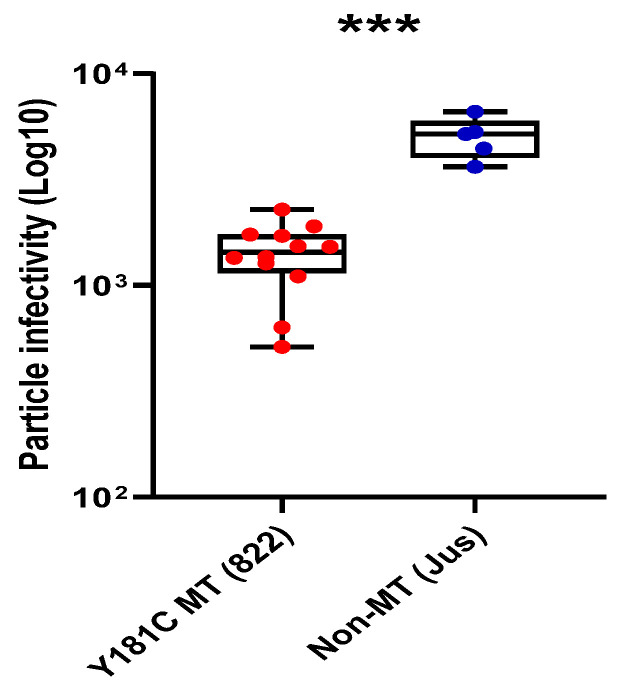
**Per particle infectivity of the Y181C mutant and non-mutant variants.** The plot displays infectivity as log_10_ values of relative light units (RLU), with each virus’s particle infectivity depicted as a dot on the box-and-whisker plot. TZM-bl cells were infected with the virus at an MOI of 0.02 with DEAE-dextran. Pairwise comparisons were executed using Mann-Whitney test. The Y181C mutant and non-mutant variants are represented as red and blue circles respectively. *** *p* value < 0.0003.

In order to compare the per particle infectivity of the mutant and non-mutant T/F clones, we performed a single-round infection assay by infecting TZM-bl cells with equal amounts of virus (0.02 MOI). We found that the infectivity of one of the 13 clones from sample 822 was very low, and it was therefore removed from all further analyses. The per particle infectivity of the Y181C mutant viruses was found to be significantly lower (*p* < 0.0003) than that of the non-mutant viruses (Figure 4). The observed reduction in per particle infectivity was in the magnitude of approximately 3.6-fold. This could again be attributed to the lower activity of the mutant RT enzyme.

### 3.6. Co-Receptor Usage

**Figure 5:**
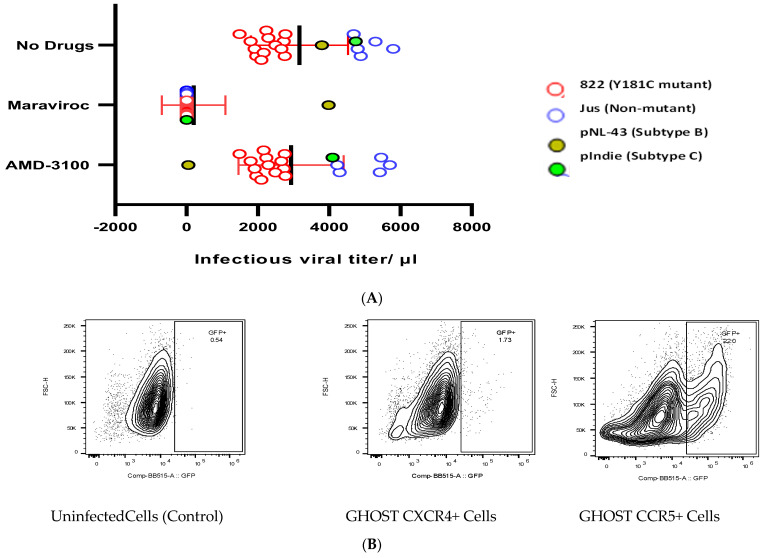
**Determination of preferred co−receptor use and tropism of the viral clones.** (**A**) The diagram represents the infectious viral titre tested in TZM−bl cells with and without the CCR5 inhibitor (maraviroc) and CXCR4 inhibitor (AMD3100) at a concentration of 2 μM. Post−infection, cells were stained with β−gal substrate and the number of blue cells were counted to determine infectivity in the presence and absence of the co−receptor inhibitors. (**B**) The expression of GFP upon infection with GHOST (3) cells (CXCR4+ and CCR5+) by both group of viruses was measured using flow cytometry. Three days post−infection, the cells were harvested and live cells were gated based on their forward and side scatter and analysed for GFP expression.

The co-receptor usage pattern of the viral clones was analysed by examining their infectivity in the presence and absence of co-receptor antagonists like AMD-3100 (that blocks CXCR4) and maraviroc (that blocks CCR5). All the viral clones tested were found to be inhibited by high concentrations of the CCR5 inhibitor maraviroc, indicating that the viruses predominantly utilized the CCR5 co-receptor for cellular entry (Figure 5A). On the other hand, none of the isolates were inhibited by high concentrations of the CXCR4 inhibitor AMD3100, providing further evidence to suggest that all the viral clones derived from recently infected individuals were R5-tropic and majorly used the CCR5 co-receptor for cellular entry, irrespective of their drug-sensitivity pattern.

The co-receptor usage pattern was further confirmed by infecting GHOST cells expressing either CCR5 or CXCR4 with the viruses and analysing the cells for GFP expression using flow cytometry. A distinct shift was observed in the cell population based on GFP expression upon infection of CCR5+ GHOST cells, while there was no observable shift in the cell population upon infection of CXCR4+ GHOST cells (Figure 5B).

### 3.7. Sensitivity to Maraviroc

**Figure 6:**
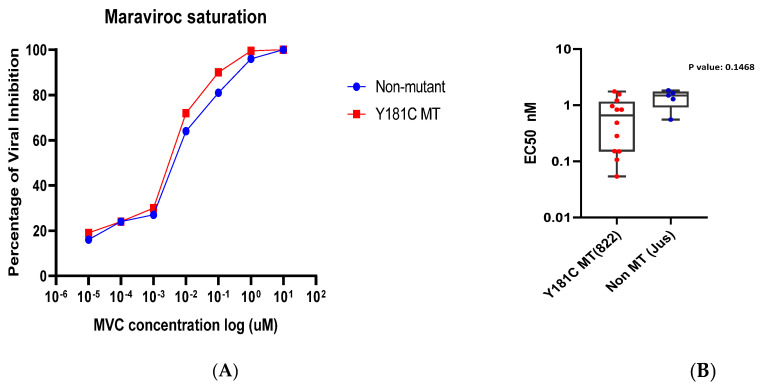
**Half−maximal effective concentration (EC_50_) of a CCR5 antagonist, Maroviroc, tested against the Y181C and non-mutant viral variants.** TZM−bl cells were infected with the Y181C mutant and non-mutant viruses at an MOI of 0.05 and cultured in the presence of the CCR5 antagonist maraviroc. (**A**) Dose–response curves for maroviroc at concentrations ranging from 10 M to 10^−5^ nM for the Y181C mutant viruses and non-mutant viruses. (**B**) EC_50_ of maraviroc against each of the Y181C mutant and non-mutant viruses. Means of the two groups were compared using unpaired *t* test and found to be non-significant (*p* = 0.1468).

We performed a single-round infection assay on TZM-bl cells in the presence of varying concentrations of the viral entry inhibitor maraviroc. For this purpose, the full-length T/F clones were exposed to MVC at concentrations ranging from 1 × 10^4^ nM to 1 × 10^−5^ nM. The median 50% effective concentration (EC_50_) was derived and compared between the two groups of viruses. It was observed that the median EC_50_ value for the non-mutant viral variants was similar for both the Y181C mutant and non-mutant viral variants (Figure 6).

### 3.8. Sensitivity to Neutralizing Antibodies

**Figure 7:**
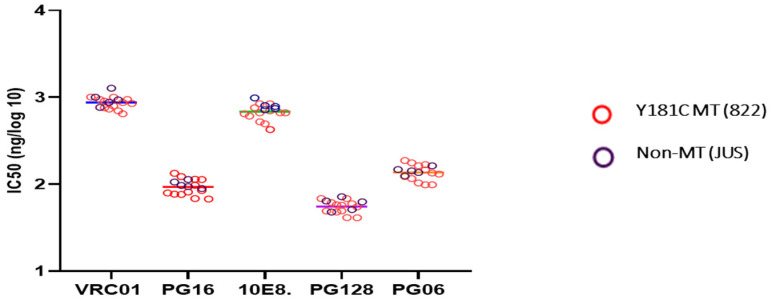
**Neutralization potential of five different monoclonal broadly neutralizing antibodies against the Y181C mutant and non-mutant T/F viruses.** The figure represents the potency of neutralization of viral isolates by bNAbs. IC_50_ represents the amount of mAbs required to neutralize half of the virus in culture.

To confirm the integrity of the viral envelopes and to ascertain the neutralization sensitivity of the viral clones, we tested the viruses against a panel of well-characterized broadly neutralizing antibodies (bNAbs) like VRC01, PG16, PG128, PG06, and 10E8. We found no significant difference in IC_50_ values for any of the tested bNAbs between the mutant and non-mutant T/F clones (Figure 7). At 1 μg/mL concentration of VRC01 antibody, the mutant variants showed 59% inhibition and non-mutant viruses showed 54% inhibition. Similarly, 10E8 gave 64% and 62% inhibition with the Y181C mutant and non-mutant viruses respectively. With PG16, PG128, and PG06, the mutant clones gave 74%, 86%, and 78% inhibition, respectively, and the non-mutants clones gave 79%, 91%, and 82% inhibition, respectively.

### 3.9. Susceptibility to NNRTIs

**Figure 8:**
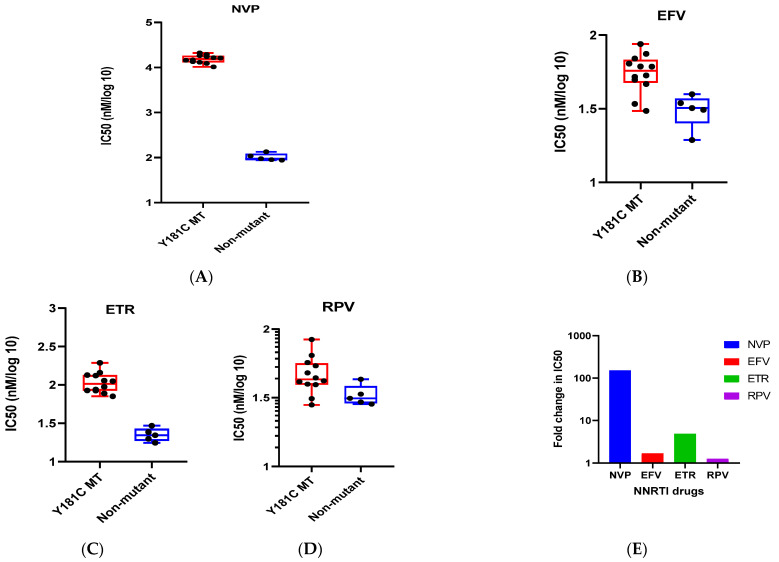
**Phenotypic resistance of viruses to the NNRTI drugs.** Phenotypic susceptibility to (**A**) NVP; (**B**) EFV; (**C**) ETR; and RPV (**D**). Fold change in IC_50_ value (**E**) as determined using a single-cycle phenotypic assay.

The susceptibility of the viruses to various NNRTIs drugs like EFV, NVP, EFV, and RPV was assessed using a single-cycle replication assay. The findings are presented in Table 3 as mean IC_50_ values and fold change. The mean IC_50_ value for NVP was found to be significantly higher for the Y181C mutant as compared to the non-mutant viruses (15,548.3 nM vs. 102.92 nM). This translated to a 151-fold (*p* < 0.0003) increase in resistance to NVP for the mutant viruses. The Y181C mutant viruses also exhibited a 4.8-fold higher resistance to ETR (IC_50_ of 110.64 nM vs. 22.64 nM; *p* < 0.0003), 1.7-fold higher resistance to EFV (IC_50_ values of 53.31 nMvs. 31.36 nM), and 1.2-fold higher resistance to RPV (IC_50_ of 42.03 nM vs. 33.16 nM) (Figure 8).

## 4. Discussion

In this study, we generated 18 full-length infectious molecular clones of HIV-1 T/F viruses from the plasma of two acutely infected infants who had acquired the infection through vertical transmission, using the single genome amplification strategy with intricate manipulations. By characterizing the T/F clones, we aimed to provide new insights into their unique biological properties. Whole genome sequencing and sequence analysis revealed very low levels of genetic diversity between the clones generated from each infant, indicating minimal viral evolution since both infants were very recently infected with the virus. Incidentally, it was noticed that the T/F viruses generated from one of the infants harboured an NNRTI drug resistance-associated mutation, while the clones obtained from the other infant did not harbour any drug resistance mutation, thus giving us an opportunity to investigate the effect of drug resistance mutations on infectivity, replication, and transmission fitness among T/F viruses. The Y181C NNRTI resistance mutation that we noticed is a commonly reported mutation occurring in newborns exposed to nevirapine either through maternal ART or through single-dose NVP prophylaxis at a frequency of 56% [37]. This mutation is known to contribute to increased rates of treatment failure in acute infection [38].

All the T/F clones belonged to subtype C and exclusively used the CCR5 co-receptor for infection. The chemokine receptor type 5 (CCR5) plays a crucial role in HIV infection, especially during the early stages of the infection process [39,40]. We found that both the drug-resistant and drug-sensitive T/F clones analysed in our study exclusively used the CCR5 co-receptor. This suggests that targeting CCR5 remains a valid therapeutic strategy even in the context of drug-resistant T/F viruses. Due to the pivotal role played by the CCR5 receptor in HIV-1 transmission, infection, and AIDS progression, extensive research has focused on developing drugs targeting CCR5 [41]. Maraviroc (MVC), a globally approved HIV entry inhibitor that blocks the CCR5 co-receptor, is now used to treat patients infected with R5-tropic HIV [42]. MVC has demonstrated substantial antiretroviral activity in a short-term clinical trial involving HIV-infected patients [43].

With the understanding that fitness costs and genetic bottlenecks constrain the transmission of viruses harbouring drug resistance mutations, we undertook precise measurement of the replicative fitness of T/F variants harbouring the Y181C mutation by infecting primary peripheral blood mononuclear cells and measuring reverse transcriptase (RT) activity in the culture supernatants at periodic time intervals. It was observed that viruses harbouring the drug resistance mutation exhibited lower RT activity, as revealed by the reduced replicative capacity (RC) score as compared to the drug-sensitive non-mutant viruses. Earlier studies that analysed the replicative fitness of drug-resistant viruses also reported diminished replicative fitness of viruses containing drug resistance mutations like Y181C/K103N and G190A as compared to wild-type viruses [13,15,44]. In contrast, some other studies reported very minimal or no impact of the single RT mutation Y181C on in vitro viral fitness [45].

We checked the per particle infectivity of the mutant and non-mutant T/F viruses and found that the per particle infectivity of the T/F variants containing the Y181C mutation was significantly lower than that of the non-mutant T/F variants, in spite of using equal numbers of infectious virus particles (MOI) for the experiment, again pointing to slower processivity of the mutant RT. Since viral infectivity is largely a feature of the viral envelope, we examined the intactness and functionality of the viral envelopes by examining co-receptor usage as well as neutralization sensitivity to some well-characterized monoclonal bNAbs. It was observed that both the mutant and non-mutant T/F clones exhibited identical co-receptor usage patterns and similar sensitivity to a panel of bNAbs, including VRC01, PG16, and 10E8, indicating the functional integrity of the viral envelopes. A mutation reversal study could have confirmed whether the observed difference in infectivity was in some way linked to the Y181 mutation, but this experiment could not be performed and therefore can be considered a limitation of this study. However, this observation has clinical implications in that it is now possible to hypothesize that drug-resistant T/F viruses remain sensitive to bNAbs and may therefore be effectively targeted by antibody-based therapies. This finding is therefore crucial for informing therapeutic strategies, particularly in populations where NNRTI resistance is prevalent due to the use of drugs like nevirapine for the prevention of mother-to-child transmission, since this mutation not only leads to decreased susceptibility to nevirapine but also offers cross-resistance to other NNRTIs, potentially impacting the efficacy of treatment. We found that the mutant viruses exhibited moderate-level resistance to etravirine (ETR) and very minimal to no effect on susceptibility to efavirenz (EFV) and rilpivirine (RPV). On the other hand, both the mutant and non-mutant viruses exhibited similar levels of sensitivity to the entry inhibitor maroviroc.

While several studies have described the deleterious effects of the acquisition of drug resistance-associated mutations on viral fitness, this is the very first attempt to characterize the impact of drug resistance mutations in T/F viruses on viral infectivity and viral phenotype. Overall, our study unveiled several interesting characteristics of early transmitted T/F viruses, those harbouring the Y181C drug resistance mutation, as well as those which did not contain any mutation. Notably, the mutant viruses exhibited diminished replicative capacity and decreased infectivity in comparison to their non-mutant counterparts. Since replicative capacity, infectivity, and viral fitness are intrinsic viral characteristics that are linked to virological and clinical outcomes, further studies in this line could help define strategies that can be effectively exploited for the early control of disease progression.

## Figures and Tables

**Table 1:** Clinical profile of the infants.

Infant ID	Mode of Transmission	Mode of Delivery	Sex/Age at Sample Collection (Days)	Diagnostic Method	Viral Load (Copies/mL)	CD4 T-Cell Count (no. of Cells/µL)	Subtype of Virus	^#^ Predicted Co-Receptor Usage Determined by G2P PhenoSeq
822	MTCT	Vaginal	M (38 days)	DNA PCR	1,231,791	384	C	CCR5 CCR5
JUS	MTCT	Vaginal	M (22 days)	DNA PCR	3,783,148	741	C	CCR5 CCR5

M, male; MTCT, mother-to-child-transmission; G2P, Geno2Pheno algorithm. ^#^ Predicted co-receptor use based on the V3 amino acid sequence (HIV Los Alamos algorithm).

**Table 2:** Primers used for cDNA synthesis, amplification, and full-length molecular clone generation.

Primer Name	Primer Sequence	Primer Sequence Used for
FLcDNA	TTTCGCTTGTACTGGGTCTCTCTAGGTAGA	cDNA synthesis for near full-length
Oligo dt 20	TTTTTTTTTTTTTTTTTTVN	cDNA synthesis for near full-length
HCFLC1F	CTTGAGTGCTCTGAGCAGTGTGTGCCCG	1st round NFL PCR
HCFLC2F	GCTCTGAGCAGTGTGTGCCCGTCTATTG	2nd round NFL PCR
HCFLC1R	AGTACAAGCGAAAAGCAGCGGCTTATAT	1st round NFL PCR
HCFLC2R	CGAAAAGCAGCGGCTTATATGCCGCATCTG	2nd round NFL PCR
LTR C F	TGGAAGGGTTAATTTACTCCAAGAA	LTR amplification
LTR C R	TGCTAGAGATTTTCCACACTACCAA	LTR amplification
Pvec F	GCGGCCGCCACCGCGGTGGAGCTCC	pIndie Vector backbone amplification
Pvec R	GCGGCCGCTCTAGAACTAGTGGATC	pIndie Vector backbone amplification
LTR Overlap vec F	TTCTAGAGCGGCCGCTGGAAGGGTTAATTTACTCCAAGAAAA	T/F LTR cloning
LTR overlap vec R	CGCGGTGGCGGCCGCTGCTAGAGATTTTCCACACTACCAAAA	T/F LTR cloning
NFL C clone F	GGTAACTAGAGATCCCTCAGACCCT	NFL cloning PCR
NFL C clone R	GGCTTATATGCCGCATCTGAGGGTT	NFL cloning PCR
5′ LTR VEC F	TTATCAAAAAGGATCTTCACCTAGATCCTT	5′ LTR _Vec fragment PCR
5′ LTR VEC R	GGATCTCTAGTTACCAGAGTCACACAATAG	5′ LTR _Vec fragment PCR
3′ LTR VEC F	TGCGGCATATAAGCCGCTGCTTTTCGCTTG	3′ LTR _Vec fragment PCR
3′ LTR VEC R	GTGAAGATCCTTTTTGATAATCTCATGACC	3′ LTR _Vec fragment PCR

**Table 3:** Inhibitory potential of non-nucleoside reverse transcriptase inhibitors (NNRTIs) against WT non-mutant and Y181C mutant viruses.

Infectious Full-Length T/F Viruses	EFV IC50 (nM)	EFV FC	ETR IC50 (nM)	ETR FC	NVP IC50 (nM)	NVP FC	RPV IC50 (nM)	RPV FC
WT—non-mutant (JUS)	31.36 ± 6.6	-	22.64 ± 4.0	-	102.92 ± 17.37	-	33.16 ± 7.8	-
Y181C Mutant (822)	53.31 ± 12.9	1.7	110.64 ± 34.1	4.8	15,548.3 ± 2994.6	151	42.03 ± 5.2	1.26

The potencies are shown as mean ± standard deviation of three replicates. EFV—efavirenz; ETR—etravirine; NVP—nevirapine; RPV—rilpivirine. FC—fold-change relative to the non-mutant.

## Data Availability

The authors confirm that the data supporting the findings of this study are available within the article.

## Supplementary Materials

The following supporting information can be downloaded at: https://www.mdpi.com/article/10.3390/v16121854/s1, Figure S1: Infectivity of IMCs in TZM-bl cells.
