# Supplementary material for: Virulence and Replicative Fitness of HIV-1 Transmitted/Founder (T/F) Viruses Harbouring Drug Resistance-Associated Mutation
Source: Viruses. 2024 Nov 29;16(12):1854. doi: 10.3390/v16121854 (PMC11680346; doi:10.3390/v16121854)
Supplement: Supplementary file 1 [file viruses-16-01854-s001.zip › viruses-3298566-supplementary.pdf]

Supplementary Figure S1: Infectivity of IMCs in TZM-bl cells

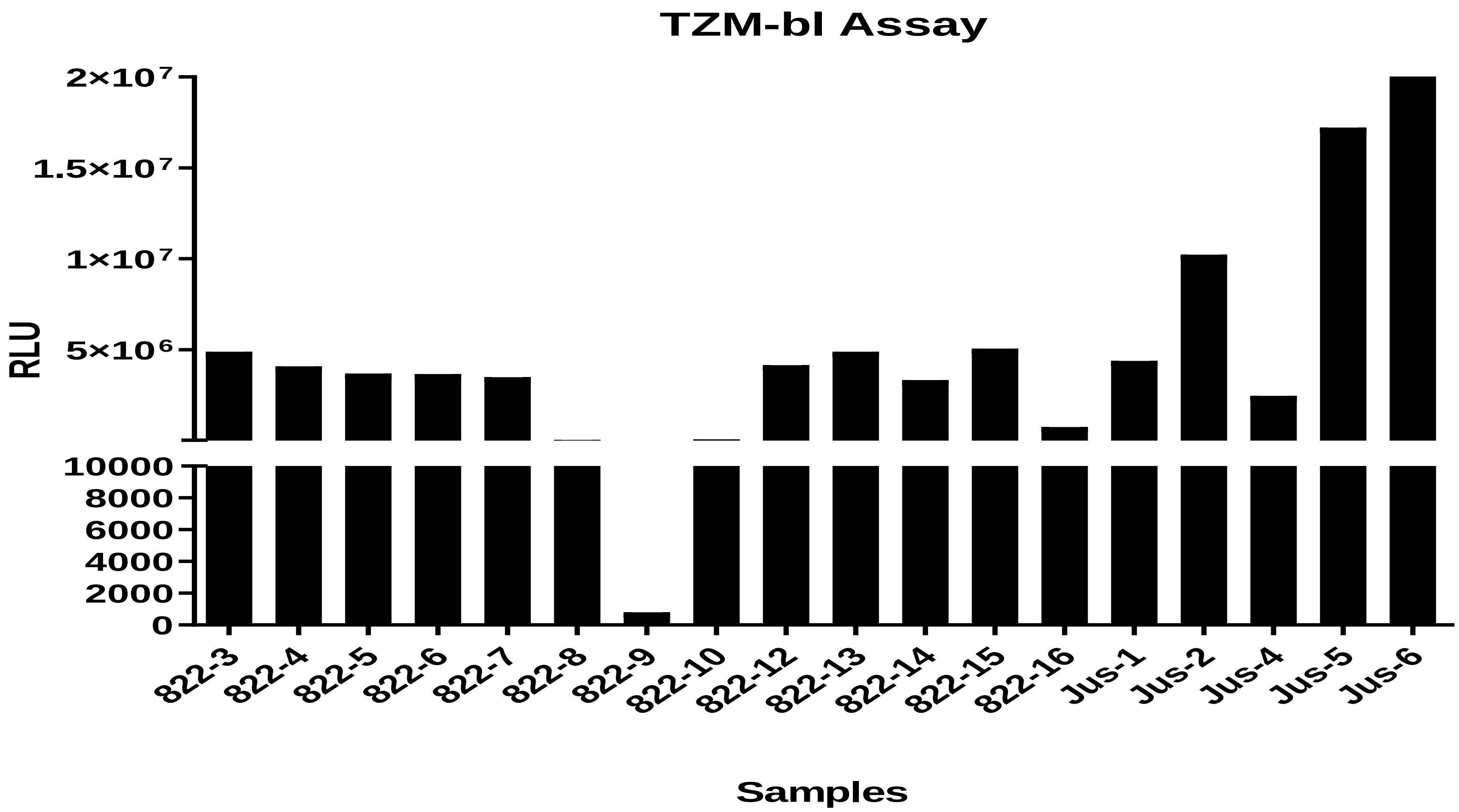

The viral clones were transfected into HEK293T cells, the virus was prepared, and the viral infectivity was estimated in TZM-bl cells using ten-fold viral dilution.
